# Comparison of sympathetic block and hemodynamic effects of erector spinae plane block and thoracic epidural analgesia in breast surgery: a randomised controlled trial

**DOI:** 10.1186/s12871-026-03694-2

**Published:** 2026-02-14

**Authors:** Gizem Kurada, Gülay Erdoğan Kayhan, Sema Şanal, Meryem Onay, Mehmet Sacit Güleç

**Affiliations:** 1 Department of Anesthesiology and Reanimation, Faculty of Medicine, Zonguldak Bülent Ecevit University, Zonguldak, Turkey; 2 Department of Anesthesiology and Reanimation, Faculty of Medicine, Eskişehir Osmangazi University, Eskişehir, Turkey; 3 Department of Anesthesiology and Reanimation, University of Health Sciences, Haydarpaşa Numune Training and Research Hospital, İstanbul, Turkey

**Keywords:** Epidural analgesia, Erector spinae plane block, Galvanic skin response, Mastectomy, Sympathetic block

## Abstract

**Background:**

In this randomized controlled, double-blind study, we aimed to investigate the involvement of sympathetic nerve fibers in erector spinae plane block (ESPB) and to compare it with thoracic epidural analgesia (TEA) in patients scheduled for unilateral mastectomy. Additionally, we compared sympathetic blockade-related hemodynamic effects, sensory blockade distribution, and analgesic effects.

**Methods:**

Thirty-eight female patients aged 18–70 years, American Society of Anesthesiologists (ASA) classification I–III, were included in the study. The patients were divided into Group E (those who received general anesthesia after the ESPB) and Group T (those who received general anesthesia after TEA). The extent of sympathetic blockade (via the hot-cold test) and skin conductance (GSR) were recorded as the co-primary outcome measures. Secondary outcomes included skin temperature, perioperative hemodynamic data, sensory block extent, postoperative pain scores, and analgesic consumption.

**Results:**

Regarding the co-primary outcomes, there was no statistically significant difference between the groups in the hot-cold test or GSR values. In terms of secondary outcomes, although the rate of skin temperature change differed (*p* = 0.028), both groups showed significant warming. Hemodynamic data were comparable. However, ESPB produced a wider ipsilateral sensory block than TEA. The postoperative pain scores, patient-controlled analgesia (PCA) demand, and rescue analgesic consumption were not statistically significantly different between the two groups.

**Conclusion:**

ESPB demonstrated similar sympathetic blockade efficacy to TEA. Additionally, it produced similar hemodynamic effects and postoperative analgesia in patients undergoing unilateral mastectomy due to malignancy.

**Trial registration:**

ClinicalTrials.gov, NCT04702061, Date of registration: 24/12/2020, https://clinicaltrials.gov/study/NCT04702061.

**Supplementary Information:**

The online version contains supplementary material available at 10.1186/s12871-026-03694-2.

## Introduction

Postoperative pain management after breast surgery is difficult due to the complex innervation of the breast and axillary region. Analgesics acting through different pathways and/or regional anesthesia techniques such as paravertebral blocks, thoracic epidural blocks, or interfascial plane blocks are commonly used [1, 2].

Thoracic epidural analgesia (TEA) remains a well-established option for thoracic and breast surgery because it provides dense and reliable analgesia [3, 4]. It continues to be favored in selected breast procedures, particularly bilateral or reconstructive surgeries, due to its proven efficacy [4]. Although interfascial plane blocks (such as serratus anterior plane block) are frequently utilized in surgeries involving the thoracic region, they primarily target the distal branches of the intercostal nerves and typically lack the spread to the sympathetic chain [1, 3]. Therefore, despite being technically more challenging, TEA is characterized by a predictable sympathetic blockade, which underlies many of its perioperative effects but may also result in hemodynamic changes such as hypotension and bradycardia [3].

Ultrasound-guided erector spinae plane block (ESPB) is an interfascial plane block that is easy to perform in the treatment of postoperative and chronic pain and is thought to have fewer side effects [5–7]. Clinical studies and reviews have demonstrated that ESPB provides effective postoperative analgesia in breast surgery, with efficacy comparable to other regional approaches [2, 4, 6, 7]. However, the mechanism of the ESPB is not yet fully understood. Imaging, cadaveric, and clinical studies suggest that local anesthetic may spread into the paravertebral or epidural spaces, potentially reaching the anterior sympathetic chain [7–10]. Based on these observations, some authors have postulated that ESPB may exert sympatholytic effects in addition to its somatic and visceral analgesic action [11, 12].

Subjective assessment of sympathetic block is based on color change, pain relief, skin temperature change, and anhidrosis. However, this assessment can be misleading, and objective tests such as skin conductance and sweat testing are recommended [13]. Skin conductance can be measured objectively with galvanic skin response (GSR) using silver electrodes placed on the skin [14]. After the sympathetic block, skin resistance increases and skin conductance decreases [13].

The aim of this randomized controlled trial was to evaluate the effects of ESPB on sympathetic nerve fibers in patients scheduled for unilateral mastectomy due to malignancy, using the hot-cold test and GSR. TEA was selected as the control group to serve as the physiological standard for sympathetic blockade [3]. The secondary aims included the assessment of skin temperature, comparison of perioperative hemodynamic parameters, the evaluation of sensory block distribution (via pin-prick test), and analgesic efficacy.

## Methods

This randomized controlled, double-blinded study was initiated after the approval of the Eskisehir Osmangazi University Clinical Research Ethics Committee (July 24, 2019; Issue No: 17) and was conducted in accordance with the principles of the Helsinki Declaration and the CONSORT guidelines for reporting randomized trials. The trial was prospectively registered at clinicaltrials.gov as NCT04702061 (Date of registration: 24/12/2020).

### Participants

Forty female patients aged 18–70 years, American Society of Anesthesiologists (ASA) physical status I–III, scheduled to undergo unilateral modified radical mastectomy or breast-conserving surgery with axillary lymph node dissection were included in the study. Exclusion criteria were pre-existing infection at the block site; contraindication to regional anesthesia; major cardiac, pulmonary, renal, and neurological disease; autonomic neuropathy or use of medications that affect autonomic function; type 1 diabetes or insulin-dependent type 2 diabetes for more than ten years; allergy to local anesthetics; cognitive dysfunction; and morbid obesity (body mass index > 35 kg/m2).

### Randomization and blinding

Patients were randomly assigned to Group E (those who underwent general anesthesia after ESPB) and Group T (those who underwent general anesthesia after TEA). Randomization was performed using a computer-generated random number table, and allocation was concealed using sealed opaque envelopes prepared by an anesthesiology nurse. For blinding, all blocks were performed by the same anesthesiologist (G.K.), who was not involved in data collection. All perioperative measurements were conducted by an independent anesthesiologist (G.E.K.), who was blinded to group allocation and the block technique.

### ESPB and TEA block techniques

All procedures were performed in a designated regional anesthesia room, which had a standard temperature (22–24 °C) and a quiet environment to ensure consistency in autonomic measurements. No premedication was administered. Patients were rested for 10 min before the procedure to allow autonomic stabilization. Electrocardiography, pulse oximetry, noninvasive blood pressure monitoring, and intravenous cannulation were performed. All blocks were performed under aseptic conditions, in the sitting position, following skin infiltration with 2% lidocaine. The same equipment and standardized protocol were used in all patients to minimize variability.

In Group E, ESPB was performed by a high frequency (3–16 MHz) linear ultrasound probe (Samsung HS50, Seoul, South Korea) guidance in the parasagittal position as described in previous studies [11, 12]. An 18 G Tuohy (Epifix, Egemen International, Izmir, Turkey) needle was inserted between the erector spinae muscle and the transverse process of the T4 vertebra using an in-plane approach. To ensure dose consistency, local anesthetics and saline were administered using separate syringes. After needle position was confirmed via hydrodissection (approximately 2 mL), 5 mL of 2% lidocaine and 10 mL of 0.5% bupivacaine were injected sequentially, followed by the remaining saline to reach a total volume of 25 mL. Following the injection, a 20 G epidural catheter was inserted 3–4 cm and fixed at the insertion site.

In Group T, T4–5 space was identified by palpation of the spinous process of vertebrae and the localization was confirmed using ultrasound in the sitting position. 18 G Tuohy needle was introduced at the T4–5 space level using the loss-of-resistance technique. After identification of the epidural space, the epidural catheter was inserted 3–4 cm and fixed at the insertion site. Following the test dose (2 mL of 2% lidocaine), 3 mL of 2% lidocaine, 5 mL of 0.5% bupivacaine, and 5 mL of saline (15 mL total volume) were administered sequentially. Finally, a 5 mL saline flush was applied to facilitate cranial-caudal spread via the volume effect [15], reaching a total volume of 15 mL. The injection site was covered with gauze and wide adhesive plaster for blinding the type of nerve block administered.

### Measurements

#### Preoperative period

**Fig. 1:**
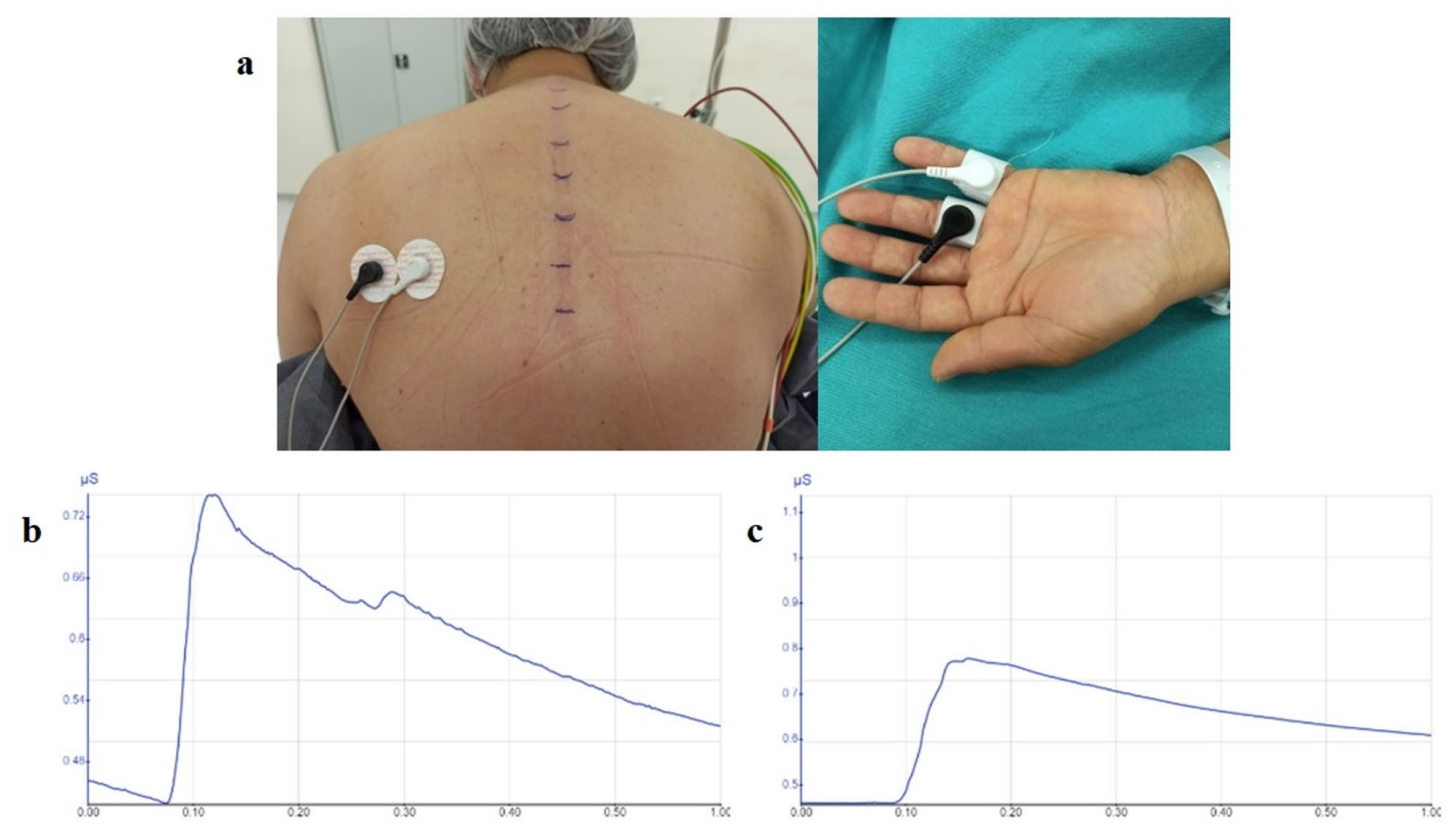
**a**. Placement of electrodes of galvanic skin response modul on the hand and on the ipsilateral thorax. **b**. Basal galvanic skin conductivity response (microSiemens). **c**. Galvanic skin conductivity (microSiemens) response obtained at the 30th min after the block was applied

In both groups, the skin conductance (GSR), skin temperature, sensory block level, heart rate (HR), and mean arterial pressure (MAP) were measured before the block (baseline value). The measurements were repeated at intervals until 45 min after the local anesthetic injection, prior to the induction of general anesthesia. Skin conductivity measurements were measured with silver electrodes using the GSR module (Neulog Galvanic Skin Response SR logger sensor NUL-217 and USB Module 200, Carolina Biological Supply Company, Burlington, NC, USA). Electrodes were placed on the volar and plantar surface of the ipsilateral hand and the ipsilateral thorax at the T4–5 vertebrae level towards the posterior axillary line (Fig. 1a). Skin conductivity measurements were performed using sympathetic stimulations created by loud auditory stimuli (abruptly playing sounds using headphones, playing screaming and crashing sounds) only at a 15 min interval after determining the baseline value to prevent habituation (Fig. 1b and c). The skin temperature from the right and left hemithorax were measured using a laser thermometer (Xiaomi, iHealth, Peking, China), and then a “hot-cold” test was performed with ethanol. The sensory block distribution area was determined using the pin-prick test at 15 min intervals.

#### Intraoperative period

Anesthesia induction was provided with 3–5 mg/kg sodium thiopental, 0.5–1 µg/kg remifentanil, and 0.6 mg/kg rocuronium bromide. Anesthesia was maintained with 2–3.5% sevoflurane (minimum alveolar concentration value 1.3) and 50% O₂-air mixture in 2 L/min fresh gas flow. When there was a 20% increase in hemodynamic parameters compared to the baseline, remifentanil infusion was started at a rate of 0.05–2 µg/kg/min. If the hemodynamic parameters were stable compared to the baseline, remifentanil infusion was not administered, and/or when there was a 20% decrease, bolus administration of ephedrine hydrochloride was planned.

Hemodynamic data (MAP and HR) before and after induction of anesthesia were recorded at 5 min intervals until the end of the operation. Total remifentanil consumption was recorded at the end of the operation. Before the extubation, 1 g paracetamol and 1 mg/kg tramadol hydrochloride were administered to both groups as a part of multimodal postoperative analgesia.

#### Postoperative period

Pain and side effects were assessed at the postoperative 1st, 4th, 6th, 12th, and 24th hour. The pain at rest and during movement was measured using a visual analog scale (VAS rest and VAS movement) (0–10 cm scale; 0: no pain, 10: worst pain). If the VAS rest score was ≥ 4 during the follow-ups, a 10 mL bolus injection of 0.125% bupivacaine was administered at 5 mL intervals from the catheter in both groups. The first injection time was recorded as the first analgesia request time. After the first local anesthetic injection, a patient-controlled analgesia (PCA) device (0.125% bupivacaine at 5 mL bolus dose, 60 min lockout time) was connected. During the evaluation, if the patient reported a VAS score of ≥ 4 even though PCA was provided, 0.5 mg/kg tramadol was planned as a rescue analgesic. At the postoperative 24th hour, the catheter was removed and the study was terminated. Total consumption of local anesthetic and tramadol hydrochloride was recorded.

### Outcome measures

The primary outcome measures of this study were the evaluation of the sympatholytic effect of ESPB using the extent of sympathetic blockade (via the hot-cold test) and GSR (hand and thoracic). TEA was selected as the control group to serve as the physiological standard for sympathetic blockade. The secondary outcome measures included skin temperature, perioperative hemodynamic parameters (HR and MAP), the evaluation of sensory block distribution (via pin-prick test), and analgesic efficacy (postoperative VAS scores, PCA demand, and rescue analgesic consumption).

### Sample size and statistical analyses

In a pilot study, the sample size calculation was based on one of the co-primary outcomes: the extent of sympathetic blockade (number of blocked dermatomes in the hot-cold test). The two-sample t-test was used to calculate the prior sample volume by utilizing the average difference in the number of blocked dermatomes between the groups (α error = 0.05). Assuming a 1-β error = 0.80, 15 patients in each group were determined. It was decided to include 20 patients in each group (40 total) to account for potential data loss.

For analyzing the data, IBM SPSS Statistics 21.0 (IBM Corp. Released 2012. IBM SPSS Statistics for Windows, Version 21.0. Armonk, NY: IBM Corp.) program was used. Continuous data were represented as mean ± standard deviation (SD), and categorical data were represented as percentages (%). Shapiro-Wilk’s test was used to examine the suitability of the data for normal distribution. The independent sample t-test was performed for cases with two groups to compare customarily distributed groups. The Mann-Whitney U test was used to compare the groups that did not conform to the normal distribution. For the assessment of GSR and skin temperature, changes over time were analyzed using a Linear Mixed Model (LMM) with baseline adjustment (ANCOVA design). For all other repeated measurements, including the extent of sympathetic blockade (hot-cold test), hemodynamic parameters, and sensory block characteristics (pin-prick test), the two-way repeated measures analysis of variance was used. Pearson’s exact chi-sq uare test was used in the analysis of the cross tables that were created. Since sympathetic blockade was evaluated using two co-primary outcomes (the hot-cold test and GSR), a Bonferroni correction was applied. A p value of < 0.025 was considered statistically significant for primary outcome comparisons. For all secondary outcomes, a value of *p* < 0.05 was accepted as statistically significant.

## Results

### Participants

**Fig. 2:**
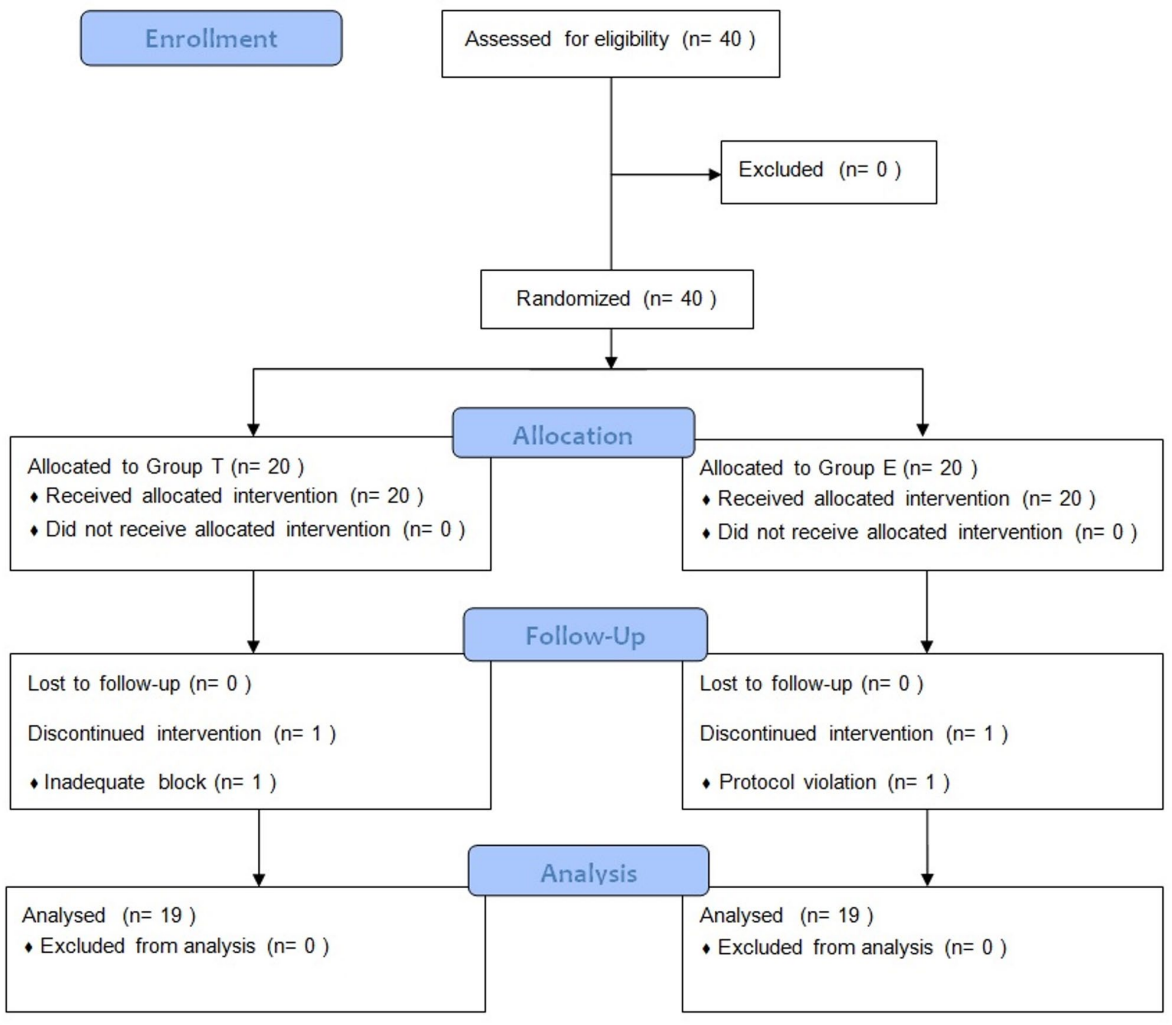
Flowchart of the study

Patients were recruited between January and March 2021, and follow-up lasted for 24 h postoperatively. The data of 38 patients were included in the analysis; one patient in Group T was excluded due to an inadequate block, and one patient in Group E was excluded due to protocol violation (Fig. 2). There were no significant differences between the groups in demographic characteristics (Table 1). The mean duration of surgery was similar in Group E and Group T (98.94 ± 35.73 min and 111.84 ± 32.32 min, respectively) (*p* = 0.20).

**Table 1:** Demographic characteristics of the patients [mean ± SD or number (%)]

	Group E	Group T	P value
Sample size, *n*	19	19	
Age (year)	52.79 ± 9.74	48.89 ± 8.09	0.13
BMI (kg/m²)	28.19 ± 3.72	27.40 ± 3.44	0.26
ASA (I/II/III)	6/12/1	5/13/1	0.94
Comorbidity	12 (63.16%)	12 (63.16%)	1.00
Diabetes	5 (26.32%)	1 (5.26%)	0.18
Hypertension	8 (42.11%)	5 (26.32%)	0.49
Coronary artery disease	1 (5.26%)	0	1.00
Neoadjuvant Chemotherapy	4 (21.05%)	5 (26.32%)	1.00

Abbreviations: *BMI* Body mass index, *ASA* American Society of Anesthesiology physical classification

### Primary outcomes

#### Hot-cold test

In the hot-cold test, no significant difference was observed between the groups on the ipsilateral hemithorax (Group × Time interaction: *p* = 0.073). It was observed that the number of blocked dermatomes in the ipsilateral hemithorax increased significantly over time when compared to the baseline in both groups (*p* < 0.001). In Group E, only one patient showed an increase in blocked dermatomes on the contralateral hemithorax – from 2 at the 15th minutes to 3 at the 45th minutes (Table 2).

**Table 2:** Number of blocked dermatomes determined by hot-cold test at ipsilateral and contralateral hemithorax [mean ± SD / min-max (median)]

	Ipsilateral		Contralateral	
	Group E (*n* = 19)	Group T (*n* = 19)	Group E (*n* = 19)	Group T (*n* = 19)
15.min	3.63 ± 2.73 0–7 (4)	1.89 ± 2.23 § 0–7 (1)	0.10 ± 0.45 0–2 (0)	2.10 ± 2.02 § 0–7 (2)
30.min	4.78 ± 2.65 § 0–9 (5)	2.78 ± 2.78 § 0–9 (2)	0.10 ± 0.45 0–2 (0)	3.47 ± 2.73 *§ 0–8 (4)
45.min	5.05 ± 3.00 § 0–10 (5)	4.36 ± 4.00 § 0–12 (4)	0.15 ± 0.68 0–3 (0)	4.36 ± 3.13*§ 0–10 (5)

* Statistically significant difference between Group E and Group T (*p* < 0.025); § Statistically significant difference in within-group comparison (*p* < 0.05)

#### GSR measurements

**Fig. 3:**
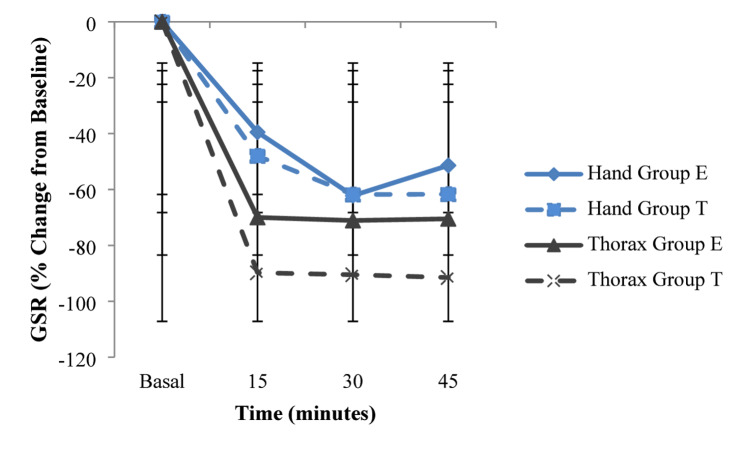
Percentage change in skin conductance (GSR) from baseline over time for hand and thoracic wall. Values are presented as mean percentage change ± standard deviation (error bars). Note: Both groups demonstrate a decrease from baseline, indicating sympatholysis. The large error bars reflect high inter-individual variability in physiological response. This finding is consistent with the repeated measures ANCOVA results which showed no statistically significant difference between groups for either site (*p* > 0.025)

To account for baseline variability, GSR measurements were analyzed using a Linear Mixed Model adjusted for baseline values. The analysis confirmed that baseline values significantly influenced the post-block measurements (*p* < 0.001). After adjustment, no significant Group × Time interaction was observed for ipsilateral Hand and Thoracic wall GSR (*p* = 0.377; *p* = 0.874, respectively). Both groups demonstrated a consistent decrease in skin conductance over time (Fig. 3).

### Secondary outcomes

#### Skin temperature

For skin temperature measurements on the ipsilateral hemithorax, both groups generally showed similar increasing trends. Statistical analysis confirmed that there was no significant difference between the groups overall (main effect of group: *p* = 0.316). However, the LMM analysis revealed a significant Group × Time interaction (*p* = 0.028), indicating a difference in the rate of temperature change between the groups. Clinically, skin temperature increased in both groups after the 30th minute. On the contralateral hemithorax, a significant increase was observed only in Group T at the 45th minute (*p* < 0.001).

#### Hemodynamic parameters

**Fig. 4:**
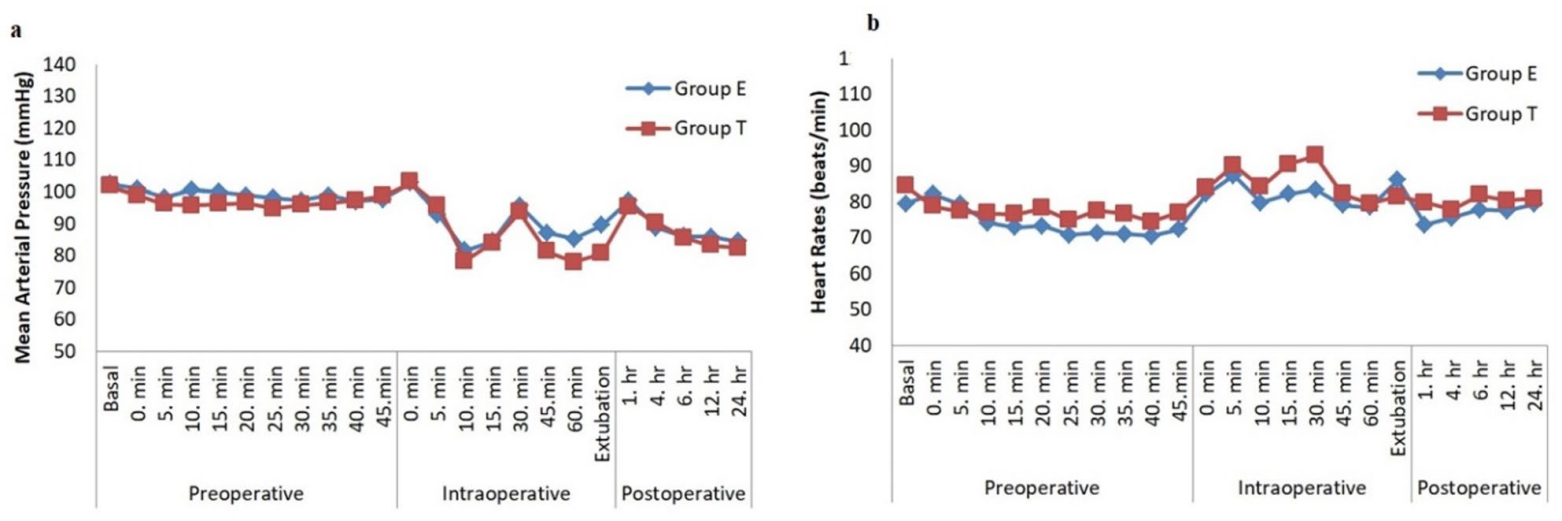
**a**. Perioperative mean arterial pressures (mmHg). **b**. Perioperative heart rates (beats /min)

There was no statistically significant difference between the groups in terms of MAP and peripheral oxygen saturation (SpO2) measurements during the preoperative, intraoperative, and postoperative periods. In the preoperative period, a significant decrease in MAP was observed only at the 25th min compared to the baseline in Group T. During the intraoperative period; a significant decrease in MAP was found at the 10th, 15th, 45th, and 60th min in both groups compared to the baseline. Ephedrine was administered to 4 (21.05%) patients in Group E and 6 (31.57%) patients in Group T due to intraoperative hypotension. In the postoperative period, MAP significantly decreased at 6, 12, and 24 h in Group E and at 12 and 24 h in Group T compared to the first postoperative measurement (Fig. 4a).

There was no significant difference between the groups in terms of preoperative HR. Within-group analysis, a significant decrease in HR was observed between 15th and 45th min in Group E, and between 5th and 45th min in Group T during the preoperative period (Fig. 4b).

#### Sensory block distribution

The dermatomal spread of the ipsilateral sensory block was significantly wider in Group E than in Group T at all-time points (*p* < 0.001). Within-group comparisons, the dermatomal extent of ipsilateral sensory block increased over time in both groups (Table 3). Contralateral sensory block was observed in four patients (21.05%) in Group E. The sensory block spread to the axillary region in eight patients (42.10%) in Group E and seven patients (36.84%) in Group T.

**Table 3:** Dermatomal spread of sensory block in ipsilateral and contralateral hemithorax [mean ± SD / min–max (median)]

	Ipsilateral		Contralateral	
	Group E (*n* = 19)	Group T (*n* = 19)	Group E (*n* = 19)	Group T (*n* = 19)
15. min	4.42 ± 2.09 *§ 0–8 (5)	1.94 ± 2.14 *§ 0–6 (2)	0.15 ± 0.50 * 0–2 (0)	2.21 ± 2.17 *§ 0–6 (2)
30. min	5.94 ± 1.43 *§ 4–9 (6)	3.15 ± 2.96 *§ 0–10 (3)	0.63 ± 1.46 * 0–5 (0)	3.26 ± 2.70 *§ 0–10 (4)
45.min	6.94 ± 1.58 *§ 4–9 (7)	3.84 ± 2.77 *§ 0–10 (4)	0.94 ± 2.22 * 0–8 (0)	4.15 ± 2.273 *§ 0–10 (4)

* Statistically significant difference between Group E and Group T (*p* < 0.05); § Statistically significant difference in within-group comparison (*p* < 0.05)

#### Intraoperative analgesic requirement

Only three patients in Group E and five patients in Group T required intraoperative remifentanil infusion. The mean remifentanil consumption rate was 0.21 ± 0.05 µg/kg/min in Group E and 0.55 ± 0.61 µg/kg/min in Group T (*p* = 0.393).

#### Postoperative analgesic efficacy

**Fig. 5:**
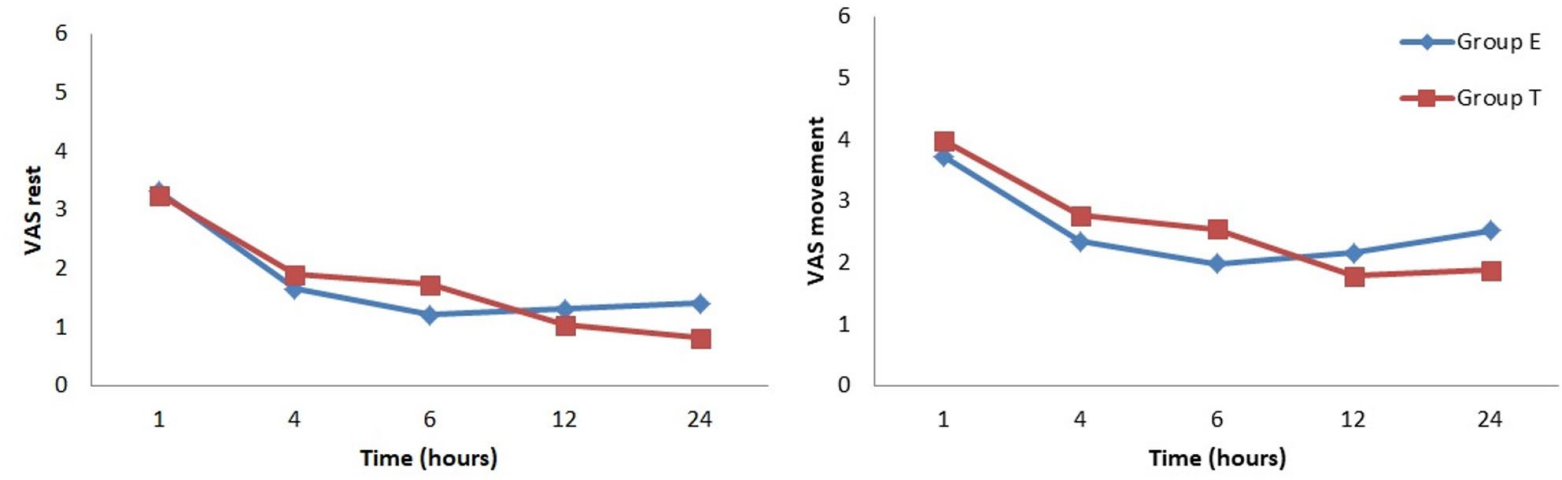
Postoperative visual analog scales (VAS) during rest and movement

There were no statistically significant differences between the groups in postoperative VAS scores at rest or during movement (Fig. 5). The number of patients who started PCA was 10 (52.63%) in Group E and 11 (57.89%) in Group T (*p* = 1.00). The first analgesic request time was 160 ± 151.65 min in Group E and 170 ± 247.31 min in Group T (*p* = 0.58). PCA demand was 18.94 ± 39.02 in Group E and 20.84 ± 43.67 in Group T (*p* = 0.87). The mean bupivacaine consumption was similar in both groups (3.58 ± 3.67 mg in Group E and 3.75 ± 4.02 mg in Group T, *p* = 0.91). Total tramadol consumption as a rescue analgesic was also similar between the groups (9.21 ± 19.73 mg in Group E, 2.36 ± 10.32 mg in Group T; *p* = 0.18).

No puncture-related complications (e.g., inadvertent dural puncture, epidural hematoma, or nerve injury) or post-dural puncture headache were observed in any patient in the TEA group.

## Discussion

In this randomized controlled study, ESPB and TEA showed no differences in sympathetic block. While a few studies have evaluated the sympatholytic effects of ESPB using semi-objective or indirect objective methods, to our knowledge, this is the first randomized controlled trial to evaluate sympathetic blockade using a direct objective method alongside the clinical evaluation of dermatomal sympathetic spread. Additionally, regarding secondary outcomes, ESPB provided similar hemodynamic effects, wider sensory block on the ipsilateral side, and comparable postoperative analgesic efficacy in terms of postoperative pain scores, PCA use, and rescue analgesic requirements.

In our study, the lack of significant differences between the groups in the co-primary outcomes (the hot-cold test scores and GSR) suggests that ESPB induces sympathetic blockade similar to TEA. Although the LMM analysis for skin temperature showed a statistical difference in the rate of warming (*p* = 0.028), both groups demonstrated a significant temperature increase, supporting the presence of sympathetic blockade. Even though current clinical practice has shifted towards fascial plane blocks, TEA remains a well-established physiological reference for sympathetic blockade. Therefore, the comparable sympathetic blockade observed in this study supports the sympatholytic efficacy of ESPB. However, as GSR measurements were performed only on the ipsilateral side, these findings primarily reflect unilateral sympathetic blockade and may not fully capture potential systemic effects. The sympatholytic effect of ESPB is an increasingly popular topic in the literature. Darçın et al. [11] observed increased internal mammary and radial artery diameters after bilateral ESPB in coronary artery bypass grafting surgery and associated this vasodilation with sympathetic blockade. Hong et al. [12] evaluated the sympatholytic effect of high-thoracic ESPB using the perfusion index (PI) and found significant increases, particularly at 10 min after blockade. Similarly, İlhan et al. [16] found increased skin temperature, PI, and optic nerve sheath diameter in patients with chronic radicular pain and associated these increases with sympathetic blockade. However, these studies assessed sympathetic blockade using semi-objective or indirect objective methods. Gungor et al. [17] stated that skin conductivity measurement methods are more successful than conventional methods in the evaluation of the sympathetic block. In our study, sympathetic block was evaluated by GSR alongside the hot-cold test and skin temperature. The GSR results in our study add objective evidence to the literature for the potential sympatholytic effect of ESPB.

Although no differences were found between the groups in MAP and HR values ​​during the preoperative, intraoperative, and postoperative periods, significant decreases were observed in the within-group analyses. Despite the publications stating that ESPB provides stable hemodynamics in the perioperative period, Sullivan et al. [18] reported hypotension due to sympatholysis [11, 19, 20]. Darçın et al. [11] found no significant changes in MAP and HR after ESPB in coronary artery bypass grafting patients. The different results from our study may be related to the volume and concentration of the local anesthetic used. The total volume used in our study (25 mL) and the addition of lidocaine may have increased hemodynamic interference. Additionally, Darçın et al.‘s measurements were limited to 45 min after the block was administered [11]. Elsabenny et al. [21] compared ESPB, TEA, and the serratus anterior plane block administered preoperatively for post-thoracotomy pain, and they reported that ESPB provided more stable intraoperative hemodynamics than TEA. While they attributed the hypotension rate of 76.5% in the TEA group to the sympathetic effects of epidural injection, they suggested that a hypotension rate of 41.2% with ESPB might be secondary to the possible spread of the local anesthetic to the paravertebral and epidural spaces. In their study [21], top-up doses were administered every 60 min in the intraoperative period after the initial dose in the TEA group and the local anesthetic concentration used in ESPB was more diluted, unlike our study. Shokri et al. [22] compared bilateral ESPB and TEA regarding minor complications (hypotension, bradycardia) in the transthoracic esophagectomy and observed less hypotension in patients receiving ESPB. Unlike our study, in the study of Shorki et al. [22], the ESPB was administered bilaterally with a lower volume (15 mL) for each side. Moreover, the injection was administered between the rhomboid major and the erector spinae muscles in the ESPB group, as Forero et al. [5] had firstly described. Giving a lower volume to the more superficial fascial plane may have reduced the possibility of hypotension by reducing the spread of the local anesthetic to the paravertebral space and, subsequently, to the epidural space.

Adhikary et al. [23] found that ESPB spreads between five and nine levels. The sensory block levels found in our study supported previous studies. Additionally, the sensory block levels detected in Group E were wider than in Group T during all preoperative measurements. One of the possible reasons for the difference in the sensory block levels may be the administration of more local anesthetic volume to the unilateral hemithorax in ESPB, whereas the bilateral spread of smaller local anesthetic volume in the epidural space in TEA. The injection of the local anesthetic via the Tuohy needle in ESPB versus the intermittent administration of the local anesthetic in TEA via the inserted catheter may also explain the difference in the sensory block levels. ESPB may be a favourable technique in unilateral breast surgeries owing to this superior block level.

In our study, there was no difference between the intraoperative and postoperative analgesic efficacy of ESPB and TEA. Ajkay et al. [4], in their study comparing opioid consumption and hospitalization after mastectomy found that, on the first postoperative day, opioid consumption was lower in the ESPB group than in the TEA and systemic analgesic groups. However, no significant difference in opioid consumption was found between the groups on the second postoperative day [4]. Leong et al. [6] reported that ESPB provides less analgesia than the pectoralis nerve block and similar analgesia without significant side effects according to the paravertebral block after breast surgery. Guan et al. [24] conducted a meta-analysis of 20 studies and showed that ESPB reduces both the intensity of postoperative pain and opioid consumption in breast cancer surgery. Elsabeeny et al. [21] observed similar pain scores and morphine consumption with TEA and ESPB after thoracotomy. Nagaraja et al. [25] compared continuous bilateral ESPB and TEA for cardiac surgery and observed similar VAS scores on the first postoperative day. Lower VAS scores were on the second postoperative day in the ESPB group [24]. Shokri et al. [22] also reported comparable VAS scores, less opioid consumption, shorter hospital stay, and higher patient satisfaction in the ESPB group after transthoracic esophagectomy. In our study, no additional local anesthetic was needed in 50% of patients in both groups on the first postoperative day, and the first analgesic demand times were similar. We found that the postoperative analgesia provided by ESPB is equivalent to that provided by TEA, similar to previous studies.

The data obtained from the hand electrodes were interpreted as cranial spread to the upper thoracic segments and anterior plane transition of the local anesthetic. Yang et al. [26] compared retrolaminar block and ESPB in cadavers and mentioned the better lateral spread of ESPB. In the review of Chin and El-Boghdadyl [27], spread to the ventral rami was considered the most likely cause in mechanism. Similarly, a recent review by Yang et al. [7] highlighted that ESPB may act through multiple mechanisms, including spreading to the paravertebral or epidural space, the posterior and anterior rami, the lateral cutaneous branches, and the sympathetic chain. However, the review emphasized that the diffusion pattern of ESPB remains variable and not fully understood [7]. We considered that the data obtained from the body electrodes also supported the spread of the local anesthetic anteriorly to the ventral rami (with lateral cutaneous involvement). In contrast, the less significant values obtained from the body electrodes were attributed to the lower number of sweat glands in the thoracic skin. The skin temperature measurements also supported the data obtained with GSR.

Although ESPB is known as a unilateral block, the possibility of creating a bilateral block crossing the midline should be considered. Tulgar et al. [28] also observed less dermatomal level of contralateral sensory block after administering unilateral ESPB for postoperative analgesia in nephrectomy. Similarly, we detected that the number of dermatomes blocked in the contralateral hemithorax was less than in the ipsilateral hemithorax in Group E. This finding suggests that the spread of the local anesthetic to the paravertebral area, even the transition to the epidural space, plays a role in the mechanism of action of ESPB. However, further studies are needed to explain the reason behind the contralateral spread that occurs in only some patients. In addition to cadaveric and clinical magnetic resonance imaging studies showing local anesthetic spread from the epidural space to the contralateral side in ESPB, case reports have documented sympatholysis and sensory block [10, 18, 19, 28–30]. Sullivan et al. [18] reported Harlequin syndrome after ESPB at the T3 vertebral level to a patient scheduled for a radical mastectomy and axillary dissection. The authors suggested that the sympatholysis seen after ESPB meant that the local anesthetic spreading to the paravertebral area blocks the rami communicantes or the sympathetic chain activity [18]. Elkoundi et al. [29] observed priapism after ESPB was administered at the L4 vertebral level due to complex regional pain syndrome in the right knee. The authors explained the priapism by the fact that the local anesthetic crossed the midline and spread to the bilateral sympathetic chain [29].

The volume of local anesthetic may contribute to this extensive spread. We used 25 mL, a volume that is consistent with the 20–30 mL range reported in thoracic ESPB applications [4, 11, 12, 21]. Since higher volumes such as 30 mL are associated with bilateral effects [28, 29], the 25 mL dosage may have facilitated spread to the paravertebral or epidural space, contributing to the observed contralateral effects. Therefore, the wider dermatomal block observed in the ESPB group compared to TEA may be related to this volume difference; the extensive spread might be facilitated by the higher volume required for this fascial plane block.

### Limitations

This study has some limitations. First, it was conducted at a single center with a relatively small sample size, which may affect generalizability of the results. Second, only female patients undergoing unilateral mastectomy were included; therefore, the results may not be applicable to other surgical procedures or male patients. Third, the preoperative evaluations were limited to 45 min after the block application to ensure standardization. Although lidocaine was used in the local anesthetic mixture to accelerate onset, this time period may have been limiting for the autonomic, sensory and hemodynamic effects. In addition, due to equipment limitations, GSR measurement could only be performed on the ipsilateral side, preventing the evaluation of potential contralateral effects. Lastly, the spread of local anesthetic to the paravertebral or epidural space was not confirmed by imaging techniques.

## Conclusion

In conclusion, no significant difference was found between ESPB and TEA regarding sympathetic block efficacy. Similarly, hemodynamic effects were comparable between the groups. Furthermore, ESPB produced similar analgesia to TEA in patients undergoing unilateral mastectomy due to malignancy.

## Supplementary Information

Supplementary Material 1.

## Data Availability

The data used and analyzed during the study are available from the corresponding author upon reasonable requests.

## Abbreviations

ASA: American Society of Anesthesiologists
ESPB: Erector Spinae Plane Block
GSR: Galvanic Skin Response
HR: Heart Rate
MAP: Mean Arterial Pressure
PCA: Patient Controlled Analgesia
SpO2: Peripheral Oxygen Saturation
TEA: Thoracic Epidural Analgesia
VAS: Visual Analog Scale

## Declerations

The study is produced from the academic thesis, also presented as an oral presentation at the 55th national congress of the Turkish Anesthesiology and Reanimation Society, Antalya/ Turkey, 28–31 October 2021.
